# *In silico *whole-genome screening for cancer-related single-nucleotide polymorphisms located in human mRNA untranslated regions

**DOI:** 10.1186/1471-2164-8-2

**Published:** 2007-01-03

**Authors:** Abdel Aouacheria, Vincent Navratil, Ricardo López-Pérez, Norma C Gutiérrez, Alexander Churkin, Danny Barash, Dominique Mouchiroud, Christian Gautier

**Affiliations:** 1 Laboratory of Biometry and Evolutionary Biology, CNRS UMR 5558, Claude Bernard University Lyon 1, 69622 Villeurbanne, France; 2 Servicio de Hematología, Hospital Universitario de Salamanca, Centro de Investigación del Cáncer, Universidad de Salamanca-CISC, Spain; 3 Department of Computer Science, Ben-Gurion University, 84105 Beer Sheva, Israel; 4 Apoptosis and Oncogenesis Laboratory, Institute of Biology and Chemistry of Proteins, IBCP UMR 5086 CNRS-UCBL, IFR 128 Biosciences Lyon-Gerland; 7 passage du vercors, 69367 Lyon Cedex 07, France

## Abstract

**Background:**

A promising application of the huge amounts of genetic data currently available lies in developing a better understanding of complex diseases, such as cancer. Analysis of publicly available databases can help identify potential candidates for genes or mutations specifically related to the cancer phenotype. In spite of their huge potential to affect gene function, no systematic attention has been paid so far to the changes that occur in untranslated regions of mRNA.

**Results:**

In this study, we used Expressed Sequence Tag (EST) databases as a source for cancer-related sequence polymorphism discovery at the whole-genome level. Using a novel computational procedure, we focused on the identification of untranslated region (UTR)-localized non-coding Single Nucleotide Polymorphisms (UTR-SNPs) significantly associated with the tumoral state. To explore possible relationships between genetic mutation and phenotypic variation, bioinformatic tools were used to predict the potential impact of cancer-associated UTR-SNPs on mRNA secondary structure and UTR regulatory elements. We provide a comprehensive and unbiased description of cancer-associated UTR-SNPs that may be useful to define genotypic markers or to propose polymorphisms that can act to alter gene expression levels. Our results suggest that a fraction of cancer-associated UTR-SNPs may have functional consequences on mRNA stability and/or expression.

**Conclusion:**

We have undertaken a comprehensive effort to identify cancer-associated polymorphisms in untranslated regions of mRNA and to characterize putative functional UTR-SNPs. Alteration of translational control can change the expression of genes in tumor cells, causing an increase or decrease in the concentration of specific proteins. Through the description of testable candidates and the experimental validation of a number of UTR-SNPs discovered on the secreted protein acidic and rich in cysteine (*SPARC*) gene, this report illustrates the utility of a cross-talk between *in silico *transcriptomics and cancer genetics.

## Background

Genetic variations contribute to the development and maintenance of complex disorders, such as cancer, through alterations in the structure and/or abundance of individual mRNA molecules. The human transcriptome could therefore be considered as a priority target in the fight against cancer. Transcript sequences represent a key source for the search of aberrantly expressed genes and for the identification of genes whose products are deregulated in malignant cells. Among these transcript sequences, Expressed Sequence Tags (ESTs) are partial single-pass sequences of cDNAs made of mRNA from a particular organ, tissue or cell line. Since cDNA libraries are generated from a wide range of cancerous and normal tissues, ESTs can be used both for measuring relative levels of gene expression [1-6], and for detecting single nucleotide differences among sequences derived from a same gene [7,8].

It is now widely assumed that human genomic DNA contains some level of polymorphism, with single nucleotide polymorphisms (SNPs) being the most common form. Owing to large-scale discovery, SNPs constitute an emerging resource for the study of genetically complex disorders such as cancer [9,10]. SNPs localized within the coding regions of genes could modify the amino acid sequence of the encoded products through non-synonymous substitutions that, in turn, may impact protein structure and function [7,11-13]. SNPs present in the untranslated regions of genes (UTR-SNPs) may rather have effects on gene expression by affecting regulatory elements or mRNA stability [14-19]. Yet, biochemical evidences as to how UTR-SNPs located in untranslated portions of mRNAs affect gene function are still scarce. Possible mechanisms for 5'-UTR include mRNA splicing interference, regulation of transcription (e.g., through methylation), translation (e.g., through internal ribosomal entry fragments), or mRNA stability [20,21]. The role of the 3'-UTR of mRNA is seen to be as important as that of the 5'-UTR in regulating gene expression. Indeed, in addition to the well-established role of the poly-(A) tail, which confers protection to the RNA molecule from degradation by exonucleases, resulting in enhancement of translation, there are a number of motif sequences within the 3'-UTR that regulate mRNA stability and translational efficiency, including the recently identified microRNA-binding sites [22,23].

In this study, we attempted to use a computational procedure to identify novel cancer markers, or polymorphisms that could influence gene expression levels in cancer cells. We decided to focus on UTR-located non-coding polymorphisms because (i) 5'- and 3'-UTR sequences are known to influence cellular steady-state levels of mRNA; (ii) polymorphisms in these sequences are accessible using EST data; (iii) potential association between UTR-SNPs and cancer phenotype is readily assessable using library features. We first detected human genetic variants located in UTR regions and associated with cancer, i.e., UTR-SNPs that are statistically over-represented in ESTs derived from cancerous libraries. We then used predictive methods to test the potential effects of the detected polymorphisms on mRNA folding and putative UTR functional elements. This report is a first attempt to use human EST databases as a source for the discovery of cancer-associated untranslated region polymorphisms at the whole-genome level. Our digital approach was combined to standard laboratory genotyping experiments to propose a set of validated variants in the secreted protein acidic and rich in cysteine (*SPARC*) gene, a key factor in cell-matrix interactions and possibly tumour aggressiveness [24-27].

## Results

We developed an EST-based pipeline to detect cancer-associated UTR-SNPs. Details about the data mining procedures are presented in Table 1.

### Pre-selection of candidates for cancer association studies

We first identified genetic variants present in untranslated regions (UTR-SNPs) of human genes using EST sequences from different libraries. Among those, we detected genetic variants associated with cancer (i.e., those that are statistically over-abundant in EST libraries derived from cancerous cells). Our predictions relied on the digital count of ESTs rather than libraries because of the frequent lack of precision concerning the origin of the source tissue(s) (both normal and tumoral) and for statistical analysis. Despite several other limitations inherent to the EST methodology (e.g. biased or limited sampling, see discussion), this whole-genome scanning strategy had the advantage of being a completely hypothesis-free approach that allowed the *ab initio *detection of cancer-associated UTR-SNPs present on EST sequences. EST-searches led to the identification of a total of 358 UTR-SNPs (on 269 transcripts) that were present at significantly (*p *< 0.01) higher allele frequencies in tumour compared to normal tissues, out of which 47 were in 5'-UTR and 311 in 3'-UTR. Some aspects, if not all, of this discrepancy could be explained by the fact that sequencing protocols generate more ESTs matching with the 3' end of genes. Our list of UTR-SNPs that potentially contribute to the cancer phenotype is summarized in Table 2 (see Additional file 1 for the complete set). With respect to the delineation of UTR-SNPs from EST data, we estimated how large the fraction of *bona fide *SNPs was expected to be after filtering using sets of verified SNPs from dbSNPs. We found that a percentage of 37.7 % (135/358) of the cancer-associated UTR-SNPs contained in our dataset corresponds to validated UTR-SNPs (see column 6 of Additional file 1). Next, three approaches were used for controlling the false discovery rate: Bonferroni and Benjamini & Hochberg multiple testing corrections, and a resampling procedure. In practical, these statistical tests provided three different magnitudes of false positive estimation that are useful indicators prior to further analysis; the Bonferroni adjustment being more conservative than the Benjamini & Hochberg method and the resampling procedure. The candidate SNPs positive after these stringent multiple testing corrections (22/358 after Bonferroni and 104/358 after Benjamini & Hochberg, n = 10,514) are highlighted in Additional file 1. By the resampling procedure, we found that 92 observed *p*-values fell below the fifth percentile of the empirical *p*-value distribution (*p *< 5.54 10^-4^).

### Association with tumour development

Our list of cancer-associated variants contains a number of genes possibly involved in the cellular capabilities that might be acquired by cancer cells [28], e.g., translationally controlled tumour protein *TCTP*, *IL-4-R*, *HLA class II antigens, TIMP-3, CD147, CD44*, and the *jun-B, c-fos, AF4, Ki-Ras *and *RAF *proto-oncogenes. Also included in our list are 38 novel sequences, i.e., entries for which no annotation was available at the time of the study (these transcripts are referred to as 'NULL' in Additional file 1). In particular, we identified a ~800 bp- long nucleotide sequence located in the 5'-UTR of ENST00000285718, which contained as many as ten cancer-associated UTR-SNPs. The corresponding gene (encoding a putative proline-rich protein) has been mapped to 2q13, a region defined as a tumor amplicon [29]. Furthermore, out of the 269 RNAs with UTR-SNPs, the screen returned 22 hits previously identified as bearing cancer-associated non-synonymous coding SNPs (nsSNPs) on the basis of a similar computer-based screen [7]. Among these transcripts exhibiting both cancer-associated nsSNPs and cancer-associated UTR-SNPs (highlighted in light grey in Additional file 1) are those encoding Heat shock cognate 71 kDa protein, polyadenylate binding protein (PABP)-3, translationally controlled tumour protein (TCTP), immunoglobulin gamma FcRIIIA, and dynein light chain 1 (DNCL1, see Table 2).

"Hot spots" for base substitutions were found for some transcripts, either as consecutive SNPs (e.g., 1286 c→a and 1287 t→c for ENST00000234617) or as 'nests' of SNPs (e.g., 991 g→t, 999t→c and 1005 c→t for ENST00000285718). However, most transcripts (~75%) displayed a unique cancer-associated SNP. We found a variant causing a g→c change at nucleotide 175 in the 5'-UTR of *RhoH*, a gene prone to aberrant hypermutation activity in lymphomas [30]. Interestingly, determination of the origin of the EST libraries revealed that this UTR-SNP was specific to lymphoid tissues. In addition to the previously reported 4 c→a and 956 t→c alterations in the 5'- and 3'-UTRs of Kruppel-like factor 6 (*KLF6*), an important DNA-binding transcriptional regulator [31], our analysis also revealed a 1206 c→t polymorphism in the 5'-UTR of this gene. Owing to the high mutation frequency of *KLF6 *in a number of pituitary tumors [32], knowledge of these *KLF6 *polymorphisms may be important for prostate cancer diagnosis.

Last, we found among the hits a series of UTR-SNPs concerning the *SPARC *gene, which encodes a multifunctional glycoprotein playing roles in tissue development, remodelling and fibrosis [24-27]. As a regulator of cell-extracellular matrix (ECM) interactions, SPARC is thought to represent a major factor in the ECM remodelling occurring during tumour invasion. Our *in silico *analysis revealed 4 UTR-SNPs located in the 3'-UTR of the *SPARC *gene, corresponding to 1474 g→a, 1551 g→c, 1922 t→g and 2072 c→t changes, which were significantly associated with the tumoral state. Noteworthy, of all the 'digital' hits, the 2072 SPARC polymorphism had the clearest association with cancer (see Table 2 and Additional file 1). This SNP is localized in a 44 bp- long conserved sequence between rodents and primates, suggesting that it might belong to a functionally constrained region.

### Detection of SPARC variants in tumour samples

Because testing every prediction in our collection would be very labour intensive, we sought to validate experimentally the predictions that were made computationally for one of the candidate transcripts. The rationale for SPARC selection was based on the following criteria: (i) multiple hits over a wide range of *p*-values; (ii) best score for one of the hit (*p*-value for 2072 c→t = 5 10^-17^); (iii) multifunctional protein; (iv) candidate for tumours with a highly invasive phenotype (i.e., with poor prognosis). A group of 18 acute myeloblastic leukemias (AML) was explored for seeking the four *SPARC *variants predicted by computational analysis (primers are listed in Additional file 2). Three of them (1551, 1922 and 2072) were detected in some of the samples while the 1474 mutation could not be detected (Table 3). In addition, a 2168 g→a change and a triple base substitution at position 2218 were identified. Allelic frequencies for each SNP in AMLs were compared with those in normal controls (n = 20): SNP 2072 and 2168 frequencies were increased in patients versus controls, although the differences were statistically significant only for the last one. Of note, the computer-based procedure failed to identify the 2168 g→a substitution because the reference *SPARC *RNA available from Ensembl (release 16.3) was only 2104-bp- long. Moreover, since our algorithm is exclusively devoted to the detection of substitutions and not of indels, the three base insertion at position 2218 also was not identified through the *in silico *screen. In any case, for the four UTR-SNPs predicted through the computer-based procedure, results from experimental validation correlated with the *p*-values obtained from the EST scanning. Moreover, this analysis indicates that the *in silico *approach presented here can help to select candidate genomic regions within which mutations can be sought.

### Patterns of substitution

In addition to a gene-centric view, SNPs can be characterized by type of nucleotide change and putative functional effect. The objective of this section was to examine the substitution patterns among the cancer-associated UTR-SNPs identified by our computer-based procedure.

We explored the distribution of the various types of simple substitution SNPs in the different sets of candidate UTR-SNPs, i.e. the complete dataset of UTR-SNPs (n = 20,304), the total pool of cancer-associated UTR-SNPs (n = 358), and the subset of UTR-SNPs which were positive after the resampling procedure (n = 92) and that are less likely to correspond to false positives. The transition rates were around 70 % and the transversion rates were ~30% in the different categories, in accordance with previous genome-wide estimates [33,34]. In all cases, the most common substitution was C→T (see Additional file 3 and Additional file 4 for a graphical representation); however, this type of change was 1.5 times less frequent in the pool of UTR-SNPs positive after the resampling procedure as in the total dataset (18.5 % *versus *27.8 %, respectively). At the same time, the T→C transition accounted for 16.3 % of all single nucleotide substitutions within this pool *versus *9.6 % within the total dataset. The couple of complementary substitutions A↔T followed a similar distribution in the total and cancer-associated datasets. Similarly, G→T and A→C frequencies were of similar magnitude in the three datasets; however, one can see that the frequencies for the complementary substitutions T→G and C→A behave in opposite manner: T→G substitutions were over-represented in the pool of UTR-SNPs positive after the resampling procedure (8.7 % *versus *4.2 % in the total sampling) whereas only ~2 % of UTR-SNPs were of type C→A in the cancer-associated datasets (*versus *4.9 % in the total pool of UTR-SNPs). Last, while the global frequencies of C↔G did not differ significantly between the different datasets (see Additional file 4, panel A), when the SNPs are reported respective of the direction of change, the frequencies of the pairs C→G and G→C showed a pattern reversal in the pool of UTR-SNPs positive after the resampling procedure compared to the total dataset (1.1 % *versus *6.3 % for C→G, and 6.5 % *versus *2.6 % for G→C, respectively). Together, these results show that the ratios of several types of substitutions differ between the entire dataset of UTR-SNPs and cancer-associated alleles.

### Possible impact of cancer-associated UTR-SNPs on mRNA secondary structure and UTR regulatory elements

Although many of the UTR-SNPs identified in our experiment are not expected to be functional, but rather to act as markers for functional variants yet to be discovered elsewhere in the gene or even possibly in a nearby gene, it is possible that at least a fraction represent functional SNPs. Therefore, we decided to assess the putative structural and functional consequences of the tumor-associated UTR-SNPs on mRNA metabolism (mRNA secondary structure and putative regulatory sites).

Sequence changes in the UTR regions can affect mRNA folding, that in turn may impact transcript stability, mRNA processing or translational control [35-40]. To assess the possible effects of our set of cancer-associated UTR-SNPs on mRNA secondary structures, we checked with computer subroutines available in the RNAMute tool [41] that are based on energy minimization methods (Vienna and MFold) [42,43] whether these changes would be predicted to induce conformational rearrangements. This program was used to compute predicted secondary structures, differences in secondary structures and corresponding free energy changes (Δ*G*) for a 100-nt window around the UTR-SNP site. 'Variant' inputs of length 100-nt were extracted from two groups of sequences: (i) sequences that displayed the cancer-associated UTR-SNPs identified through the computer-based procedure; (ii) randomly chosen sequences displaying UTR-SNPs that were not associated with the tumoral state. For each group, 'Reference' inputs were also generated from the corresponding normal allele sequences. Table 4 gives the results of variant to reference comparisons (n = 358) for the cancer-associated pool and for 10 different control datasets. Our data reveals a slight trend for cancer-associated SNPs to be found in higher distances than control SNPs. Notably, this trend becomes statistically significant (Two Sample T-test; *p *< 0.05) when only the cancer-associated SNPs positive after the permutation test (n = 92) are being considered. Among these cancer-associated UTR-SNPs, 41 (44.6%) were predicted to have no or a minor effect on RNA secondary structure (dist < 10), 29 (31.5%) were predicted to induce significant conformational changes in the folding (distance values between 10 and 50) and 22 (23.9%) were predicted to lead to high distance values with respect to their reference alleles (dist > 50) (see Table 2 and Additional file 1). In only 31.5 % of the cases (29/92) the reference allele displayed the highest negative energy value, suggesting that the majority of cancer-associated UTR-SNPs lead to more stable transcripts. However, this result should be balanced by the fact that UTR-SNPs associated with mRNA stabilizing structures have higher chance to be detected than those associated with degrading elements. The cancer-associated mutation which was predicted to cause the greatest change on mRNA structure is a c→t polymorphism on ENST00000206380 (distance = -84 using Vienna's RNADistance), a transcript that shares no similarity with any sequence in public databases. The 1551 and 2072 SNPs on *SPARC *were predicted to have a positive effect on mRNA stability (with distances of + 56 and + 38, respectively) while the 1922 polymorphism had only a mild predicted impact (distance = + 4).

Next, putative UTR functional elements potentially affected by cancer-associated SNPs were searched for using UTRscan [44]. Most of the cancer-associated polymorphisms did not lie within or at the immediate vicinity of *cis*-regulatory elements (see Table 5 and Additional file 1). A fraction of 153 UTR-SNPs out of 358 (42.7%) had an assignment to known UTR regulatory regions. When only the 92 hits positive after the permutation test are considered, the percentage of polymorphisms predicted to impact UTR functional elements remains relatively constant (37/92, i.e., 40.2 %). As shown in Table 5, a total of 9 regulatory elements out of the 31 included in the UTRSite database were located near or at cancer-associated SNP sites. Based on the UTRScan analysis, sequences close to or containing Internal Ribosomal Entry Site (IRES) elements were identified as preferential targets for cancer-associated polymorphisms, which is expected since this class of elements is the most abundant in our UTR-SNP dataset (first column of Table 5). Interestingly, a number of cancer-associated variant sequences displayed potential regulatory elements (IRES, 15-LOX-DICE) that were not apparent in the reference allele sequences. Inversely, some UTR functional elements (IRES, 15-LOX-DICE, TOP, Brd-Box and SECIS-2) were detected only in reference allele sequences but not in variant ones. Thus, *cis*-acting regulatory elements may be gained or lost when reference allele sequences are modified by cancer-associated SNPs. Loss of a SECIS-2 (for selenocysteine insertion sequence) regulatory element in the 3'-UTR of ENST00000288332 may be particularly relevant. Indeed, out of the 20,304 UTR-SNPs included in our dataset, only 12 were mapped to untranslated regions containing SECIS-2 elements. ENST00000288332 encodes a putative glutathione peroxidase, i.e., a selenoprotein, and SECIS elements are required for the translational incorporation of the unusual amino acid selenocysteine in these enzymes [45,46]. Last, two physically close cancer-associated SNPs (3726 a→g and 3743 c→t) resulted in supplementary regulatory elements (IRES and LOX-15-DICE, respectively) in the 3'-UTR of *brain-type glycogen phosphorylase*, a proposed biomarker of gastrointestinal tumours [47,48].

Altogether, these results provide evidence that at least a subset of cancer-associated SNPs might have functional consequences on mRNA stability and/or expression.

## Discussion

Owing to advances in biotechnology and bioinformatics progress, researchers can now capture "molecular portraits" of various particular cancers using gene chips or SAGE data. These methods provide information on tens of thousands of genes simultaneously, and some variations in genes might be directly related to the cancer phenotype. Transcriptome analysis not only gives information about gene expression levels in normal versus cancer cells, but also about genetic variations. In that respect, large-scale scanning of EST databases have previously been used for identification of SNPs in genes involved in a various number of disorders [49-51]. As noted elsewhere [8,9,15,52], EST-based strategies have inherent limitations, including poor sequencing depth, variations in library sizes, poor quality annotation and differences in transcript sampling. Moreover, large-scale computational studies may be hampered by artifacts produced during EST library preparation, e.g. uncertainty concerning the origin of the samples or use of pools of different cell types. With these caveats in mind, in this study, we made the assumption that UTR-SNP profiles may help to propose novel molecular signatures in cancer. Using a novel computational strategy, a set of ~350 UTR-SNPs presumably associated with the cancer phenotype was identified, and then characterized using bioinformatics tools. This list contains novel markers as well as candidate SNPs that could alter both mRNA stability, i.e., transcript abundance, and translational regulation of cancer-associated genes, i.e., protein levels. Because some UTR-SNPs may affect transcript and protein abundance, their knowledge could somehow bridge a gap between differential gene expression studies and cancer phenotype evidences. Hence, a prolongation of our study is the determination of UTR-SNPs that correlate with aberrant gene expression in cancer cells. As novel UTR regulatory sites are identified and more methods are developed to analyze mRNA secondary structure, future plans may include development of integrated and large-scale computational tools to predict UTR-SNPs with potential phenotypic consequences. Once these computational tools will be made available, it will be of interest to determine if the proportion of UTR-SNPs predicted as deleterious increases at low allelic frequencies, mirroring previous studies that were focused on nsSNPs [50,53,54]. While out of the scope of this cancer-oriented study, other genome-centric approaches may be useful such as examination of base composition around the UTR-SNP position, exploration of neighbouring-nucleotide effects, or functional annotation of the variant transcripts.

Determination of the allele frequencies for several UTR-SNPs and study of the haplotype structure of some of the loci would also likely constitute profitable avenues of research. In that respect, one of the testable hypotheses of our work is related to DNCL1. This gene encodes a highly conserved multifunctional protein known to play important roles in a variety of processes including cell proliferation, apoptosis and cytoskeleton organization, and whose deregulation could influence tumour progression [55-61]. We have recently identified and experimentally characterized a DNCL1 tumour variant (corresponding to a Gly to Cys substitution at amino acid position 79) [7], and we report here an UTR-SNP located in the 5'-UTR of the DNCL1 transcript (introducing a t→c change at position 45, see Additional file 1). The G79C mutation was shown to induce a clear conformational change to DNCL1 and to reduce substantially the *in vitro *target binding capabilities compared to the wild-type version [7]. As the possibility exists that the 5'-UTR polymorphim may be in linkage disequilibrium with the G79C mutation, it will be interesting to investigate both polymorphisms in samples from healthy and diseased donors.

Although potential UTR-SNPs relevant for cancer association studies could be successfully identified through innovative computer-based procedures, it is worth stressing that the candidate SNPs should be verified through experimental methods such as RT-PCR, microarrays and genotyping experiments, as described here for the polymorphisms located on *SPARC*. *SPARC *is a gene involved in a number of diseases including rheumatoid arthritis, scleroderma, tumor development and metastasis [62-67]. Our computer-based screen revealed four UTR-SNPs located in the 3'-UTR of *SPARC *(1474 g→a, 1551 g→c, 1922 t→g and 2072 c→t) that were significantly associated with tumor libraries. Out of these four UTR-SNPs, three were confirmed in tissue samples (1551, 1922 and 2072) and one was experimentally validated as cancer-associated in AML samples (2072). During the course of the study, two additional cancer-associated polymorphisms were discovered through the genotyping experiments (2168 g→a and a 3-bp insertion at position 2218). Interestingly, a distinct polymorphism within the SPARC gene, namely 998 c→g, has been associated with susceptibility to and clinical manifestations of scleroderma [68]. Therefore, SPARC genetic polymorphisms may represent useful candidate SNPs for screening either susceptibility to cancer (2072 c→t and 2198 g→a) or scleroderma pathogenesis (998 c→g). Moreover, recent studies have reported increased risk of cancer in patients with scleroderma [69]. Although underlying explanations are still lacking, one possibility is that alterations in SPARC could represent a common risk factor. In this hypothesis, it is noteworthy that the 1922 t→g UTR-SNP present on SPARC has been associated with scleroderma [68], in addition to cancer (our screen). In conclusion, knowledge of SPARC polymorphisms could provide potential candidate UTR-SNPs for both diseases, either separately or in combination. Last, it will be worth testing experimentally whether the identified UTR-SNPs affect gene expression. In addition to relative quantification of allelic expression by quantitative RT-PCR or Western blotting on human samples with different genotypes, functional evaluation will require demonstration of allele-specific effects on mRNA expression or stability. This can be addressed through nuclear run-on experiments and mRNA half-life studies, and construction of chimeric genes encoding the luciferase reporter sequence with the wild type or the mutated alleles. Information derived from post-genomic bioinformatics when combined to laboratory observations has the potential to greatly increase our understanding of the role of polymorphisms involving untranslated regions in disease pathogenesis.

## Conclusion

In the search for non-coding genetic variation associated with cancer, no systematic attention has been paid so far to the changes that occur in untranslated regions of mRNA. This work is a first, genome-wide attempt to identify UTR-SNPs (and flanking sequences) to prioritize for further studies in the field of cancer biomarker research. Computational analysis suggests that a proportion of cancer-associated UTR-SNPs may have the potential to significantly affect mRNA secondary structure and/or functionally important RNA regions. The *in silico *approach described here therefore sets the stage for the next phase of characterization of UTR-located functional variants in human cancer.

## Methods

### Data preparation

We have used an EST-based pipeline to scan for UTR-located polymorphisms associated with libraries of cancerous origin. Human ESTs from dbEST [70] (October 2004 release) were first extracted using the ACNUC sequence retrieval system [71]. ESTs were classified according to their UNIGENE library features [72] as previously described [6]. The eVOC ontology [73] (October 2004) for anatomical sites and pathology types was then used to classify the libraries through a number of criteria such as tissue origin and pathological context including tumor state. This well-accepted hierarchical vocabulary provided us with a mean to determine when a specific tissue was part of an organ and when a specific label was part of the 'tumoral' state. A total of 5135 'tumor' and 2503 'normal' (i.e. non-pathological) libraries were catalogued. Our approach to EST clustering used the human genome as a reliable guide. ENSEMBL RNAs [74] annotated on human genome assembly (release 16.3) were used as a backbone for the clustering of dbEST sequences using MEGABLAST (alignment length ≥ 100 bp and similarity ≥ 95%) [75]. In order to avoid paralogous false positive assignation, only best EST hit matches were subsequently selected. RNA clustering of ESTs in both normal and tumoral tissues was the starting point for *in silico *mining of UTR-SNPs associated with tumoral phenotype.

### SNP detection

We have developed an algorithm to identify exonic SNPs in multiple alignments of various ESTs associated to a particular annotated transcript. This algorithm takes advantage of EST library redundancy and performs four filters to reduce the effect of sequencing inaccuracies at each position. The first filter required that each position within a multiple alignment of ESTs should have an exact match with the reference RNA (windows length = 10 bp around each variant position). The second filter considered a position as informative if the number of libraries in the multiple alignment was superior to a fixed minimum threshold (library number ≥ 5). The third filter of the algorithm required the variant to be found at least two times independently i.e., in two different libraries. A last independent filter that required a minimum of two variant ESTs in one of the libraries was subsequently added in order to increase further the stringency of the cancer association mining strategy. We then combined detection method information (library and EST depth coverage) and nucleotide substitution features (e.g., transition/transversion, position in 5'- or 3'-UTR) for the UTR-SNPs that have been filtered out. Statistical analysis was performed using R [76]. Genomic data were stored in a local PostgreSQL database (GeMCore) [77] using PERL and Java script.

### Cancer association

Finally for each informative SNP that has both normal and tumoral EST coverage, an exact Fisher's test was performed in order to statistically evaluate the association of a particular variant with the tumoral state. We privileged the counting of ESTs rather than a count per library because of the frequent lack of precision concerning the origin of the source tissues and the use of pooled samples. To adjust *p*-values produced by the Fisher's exact test, three approaches were used: (a) Bonferroni, and (b) Benjamini and Hochberg corrections, which are very conservative methods for controlling the false discovery rate, and (c) a resampling procedure. The standard Bonferroni correction multiplies the uncorrected *p*-value by the number of statistical tests. The Benjamini and Hochberg correction consists of ranking all *p*-values and adjusting each by multiplying by the total number of tests and dividing by the rank of that *p*-value. The resampling procedure simulates the distribution of the minimum *p*-value that we would expect if there was no association with cancer. To do this, reference and variant margins were fixed at each SNP; Fisher's exact test was then performed for 1,000 resampled datasets, and the smallest *p*-value was recorded. This resampling procedure was repeated for n = 10,514 SNPs, from which an empirical distribution of the minimum *p*-value was obtained. From this distribution, we estimated the *p*-value that corresponded to the conventional 5% threshold. The intensity of the bias of tumoral versus normal allele frequency was calculated according to the following formula:

*Ib *= (*a*/*V*) - [(*T *- *a*)/*R*], where 'a' is the number of tumoral variants, 'V' the total number of variants, 'T' the sum of tumoral counts (variant plus reference) and R the total number of reference alleles (Ib being close to 1 in case of strong association).

### *In silico *characterization of UTR-located SNPs

UTRScan [78] was used to identify putative *cis *acting elements patterns in the regions containing cancer-associated SNPs. UTRScan looks for UTR functional elements by searching through user submitted query sequences for the patterns defined in the UTRsite collection [44]. To test the potential effects of the detected polymorphisms on mRNA folding, we took advantage of the RNAMute application [41].

### *In vitro *detection of SPARC variants

Genomic DNA was extracted from bone marrow in patients with acute myeloblastic leukemia (AML) and peripheral blood in healthy donors. The phenol/chloroform method was used for DNA extraction according to standard procedures. Primers to explore SPARC polymorphisms detected by computational procedures were designed based on the DNA sequence from GenBank (entry: BC072457). The sequences of the primers are listed in Additional file 2. Amplification was carried out in an iCycler Thermal Cycler (Bio-Rad, Hercules, CA, USA): 1 μg of DNA was amplified in a 25 μl of final reaction volume containing 10 × buffer II, 2 mM MgCl2, 0.2 μl of 25 mM dNTP's mixture, 0.4 μl of 20 pmol/μl forward and reverse primers and 0.2 μl of Fast-Taq polymerase (5 u/μl) (Roche Diagnostics, Indianapolis, IN, USA). PCR procedure consisted of 35 cycles of denaturation at 94°C for 15 sec, annealing at 60°C for 60 sec, with an initial denaturation of the DNA at 94°C for 5 min before PCR, and a final extension at 72°C for 5 minutes. The PCR products were sequenced in an ABI PRISM 3100 Genetic Analyzer (Applied Biosystems, Foster City, CA, USA).

## Authors' contributions

AA designed the study, analyzed the data and drafted the manuscript. VN developed the algorithm for the differential display procedure, participated in the data analyses and reviewed the manuscript. NG and RLP performed the genotyping experiments. DB and AC carried out the RNA secondary structure mutation predictions and reviewed the manuscript. DM and CG provided funding and supervision for the work. All authors read and approved the final manuscript.

## Supplementary Material

Additional File 1 Complete list of cancer-associated UTR-SNPs. UTR-SNPs with significantly different allele frequency in normal versus tumoral tissues (exact Fisher's test; *p *< 0.01). Hits are ranked by decreasing p value (see Method section). Sequences for which no annotation is available are referred to as 'NULL'. Bias intensity is given for information. Candidate positive after the multiple testing corrections are underlined. Information concerning the SNPs present on SPARC appears in bold. References appear for validated SNPs from dbSNP (last column). Ambiguity code: R = G↔A; M = C↔A; K = G↔T; Y = C↔T; S = G↔C; W = A↔T. Nucleotide sequence of the reference RNA is shown within a 10-bp interval around each SNP site. Putative *cis *acting elements located in the regions containing cancer-associated SNPs were identified with UTRScan (see text for details). RNAMute was used to compute distances between variant and reference alleles. Gene symbols were from HGNC (HUGO Gene Nomenclature Committee). UTR-SNPs lying on transcripts for which non-synonymous SNPs were previously identified [7] are marked in light grey (first column). Click here for file

Additional File 2 Polymerase chain reaction primers for detecting SPARC UTR-SNPs. Click here for file

Additional File 3 Percentages of the different types of simple substitution SNPs. Click here for file

Additional File 4 Distribution of the different types of simple substitution SNPs (graphical representation). (A) Substitutional patterns observed among UTR-SNPs. Transition rates were 67.2 % in the complete dataset of UTR-SNPs, 72.6 % in the total pool of cancer-associated UTR-SNPs, and 66.3 % in the subset of UTR-SNPs which were positive after the resampling procedure. Of the 358 cancer-associated UTR-SNPs, 260 were transition events while 298 were transversion events. When considering the 92 UTR-SNPs positive after the resampling procedure, 61 were transition events and 31 were transversion events. (B) The proportions for each pair of complementary substitutions are graphed next to each other for ease of comparison. Student t-test not significant (*p *> 0.05). Click here for file

## Figures and Tables

**Table 1:** Overview of the EST-based data mining strategy.

Total SNPs (after algorithm filtering)	~51,760
Total UTR-SNPs	20,304
UTR-SNPs with *p *< 0.05	1354
UTR-SNPs with *p *< 0.01	358
Total SNPs in 5'-UTR	47
Total SNPs in 3'-UTR	311

Identification of tumor-associated polymorphisms located in human mRNA untranslated regions. UTR-located SNPs are referred to as UTR-SNPs. SNP counts in each analytical step.

**Table 2:** Summary of cancer-associated UTR-SNPs.

Description	SNP ID	UTR	Variation			*P *value	mRNA secondary structure distance	Putative functional element
SPARC precursor [P09486/ENST00000231061]	rs1059829	**3'**	**c**	**2072**	**t**	***4,67E-17***	38	
Histidine triad nucleotide-binding protein 1 [P49773/ENST00000304043]		3'	t	483	g	***5,00E-17***	62	IRES
Ig alpha-1 chain C region [P01876/ENST00000251006]		3'	g	1643	a	***8,27E-17***	78	IRES
Annexin A1 [P04083/ENST00000257497]	rs3739956	5'	a	58	g	***1,47E-12***	8	
Lithostathine 1 alpha precursor [P05451/ENST00000233735]		3'	c	718	t	***1,17E-11***	0	
26S proteasome-associated pad1 homolog [NM_005805/ENST00000263639]	rs9713	5'	a	460	t	***3,87E-10***	12	
Glucagon precursor [P01275/ENST00000233604]		3'	g	698	a	***8,16E-09***	6	IRES *
mitochondrial ribosomal protein L41 [NM_032477/ENST00000332183]	rs698148	3'	g	526	c	***1,92E-08***	6	IRES
Actin, cytoplasmic 2 (Gamma-actin) [P02571/ENST00000331925]		3'	g	1572	a	***5,92E-08***	0	IRES
Biglycan precursor [P21810/ENST00000331595]		5'	g	94	t	***6,45E-08***	4	15-LOX-DICE
Mitochondrial processing peptidase alpha subunit [Q10713/ENST00000298536]	rs7628	3'	a	1933	g	***8,04E-08***	68	ADH_DRE
Beta-2-glycoprotein I precursor [P02749/ENST00000205948]	rs6933	3'	c	1090	t	***5,35E-07***	0	
SH3-containing GRB2-like protein 1 [Q99961/ENST00000269886]		3'	c	2116	t	***1,18E-06***	38	
dynactin p62 subunit [NM_016221/ENST00000255263]		3'	c	3575	t	**5,57E-06**	26	IRES
Interleukin-4 receptor alpha chain precursor [P24394/ENST00000170630]	rs8832	3'	a	3135	g	**1,12E-05**	52	
Ran GTPase [P17080/ENST00000254675]		3'	g	900	a	**1,32E-05**	0	IRES *
Chemokine-like factor super family member 6 [Q9NX76/ENST00000205636]		3'	t	3549	a	**1,44E-05**	14	
60S ribosomal protein L29 [P47914/ENST00000294189]		3'	c	613	a	**1,92E-05**	4	
14-3-3 protein zeta/delta [P29312/ENST00000297569]	rs11551356	3'	t	2351	c	**2,02E-05**	0	
TC4 protein [Q96QB7/ENST00000316561]		3'	g	841	a	**5,15E-05**	48	
Metallothionein-IE [P04732/ENST00000306061]	rs708274	3'	g	209	t	**5,30E-05**	66	
Retinoic acid- inducible E3 protein [Q13571/ENST00000294507]	rs1050739	3'	g	2125	a	**7,38E-05**	12	IRES *
Voltage-dependent anion-selective channel protein 2 [P45880/ENST00000298468]	rs11543	5'	g	51	c	**9,91E-05**	64	
PDZ and LIM domain protein 1 [O00151/ENST00000265995]	rs1049989	3'	t	1193	g	**1,17E-04**	0	
Pituitary tumor-transforming protein binding factor [P53801/ENST00000330938]		3'	c	2723	t	**1,33E-04**	8	
Mitochondrial import receptor TOM22 homolog [Q9NS69/ENST00000216034]	rs1056691	3'	t	966	g	**1,44E-04**	48	
Ubiquitin-like protein SUMO-1 conjugating enzyme [P50550/ENST00000219558]	rs7302	3'	t	1024	g	**1,55E-04**	16	IRES
Dynein light chain 1 [Q15701/ENST00000242577]		5'	t	45	c	1,73E-04	22	
20 kDa nuclear cap binding protein [P52298/ENST00000321256]		3'	a	1683	c	**1,80E-04**	0	
Cytochrome P450 1B1 [Q16678/ENST00000260630]	rs162549	3'	a	4412	t	**1,84E-04**	6	
PTD012 protein [NM_014039/ENST00000332038]		3'	a	2118	g	**1,98E-04**	74	
Small proline-rich protein 3 [Q9UBC9/ENST00000295367]	rs1134220	3'	t	958	g	**2,14E-04**	68	
Large neutral amino acids transporter small subunit 2 [Q9UHI5/ENST00000316902]		3'	c	2689	t	**2,25E-04**	0	
Transforming protein p21A Ki-Ras [P01116/ENST00000311936]		3'	t	1260	c	**2,51E-04**	10	IRES
nucleolar protein family A, member 3 [NM_018648/ENST00000328848]	rs1045238	3'	g	327	c	**3,07E-04**	58	TOP
Heterogeneous nuclear ribonucleoprotein K [Q07244/ENST00000297818]	rs167203	5'	c	156	g	**3,50E-04**	10	
RECS1 protein homolog [Q969X1/ENST00000258412]		3'	g	2920	a	**3,70E-04**	0	
G1/S-specific cyclin D2 [P30279/ENST00000261254]		3'	a	6471	c	**3,78E-04**	20	
Zinc finger protein 384 [Q8TF68/ENST00000319770]	rs6786	3'	t	3145	c	**4,32E-04**	0	
Myosin regulatory light chain 2 [P19105/ENST00000217652]	rs7811	3'	a	1107	g	**4,68E-04**	22	
Neuron specific protein family member 2 [Q9Y328/ENST00000303177]	rs4457100	3'	a	1030	g	**5,46E-04**	8	
Sorting nexin 4 [O95219/ENST00000251775]		3'	t	2178	a	**5,63E-04**	ND	IRES *
Paired amphipathic helix protein Sin3b [O75182/ENST00000248054]	rs1044880	3'	c	4888	t	**6,27E-04**	ND	15-LOX-DICE
FK506-binding protein 1A [P20071/ENST00000262925]		3'	a	518	g	**6,30E-04**	ND	
Epsin 4 [Q14677/ENST00000296951]	rs254682	3'	t	3136	c	**6,86E-04**	ND	
40S ribosomal protein S5 [P46782/ENST00000196551]		5'	c	27	t	**6,94E-04**	ND	
Rho-related GTP-binding protein RhoH [Q15669/ENST00000303700]	rs2245466	5'	g	175	c	**6,99E-04**	ND	
Death-associated protein. [Q9BUC9/ENST00000230895]	rs267927	5'	t	163	c	1,69E-03	ND	
Inhibitor of apoptosis protein 1 [Q13489/ENST00000263464]		5'	t	539	g	4,88E-03	ND	
Kruppel-like factor 6 [Q99612/ENST00000173785]		3'	c	1206	t	9,50E-03	ND	

The Table shows a selection of 50 UTR-SNPs (out of 358) with significantly different allele frequency in normal versus tumoral tissues (exact Fisher's test; *p *< 0.01). UTR-SNPs are ranked by decreasing p-value. Swissprot protein accession references and Ensembl transcript accession references are indicated between brackets. Candidate positive after the multiple testing corrections are set in italics (Bonferroni), in bold (Benjamini and Hochberg) or underlined (candidate positive after a resampling procedure). Predictive effect of the polymorphisms on RNA secondary structure and putative UTR functional elements is indicated. Asterisk means that the reference allele sequence is modified by the cancer-associated UTR-SNP. For full data access, see Additional file 1. Accession numbers and SNP rs numbers are indicated in column 1 (description) and column 2 (SNP ID), respectively.

**Table 3:** Results of *SPARC *genotyping analysis in AML samples.

		AML patients	Healthy donors	OR (95% CI)
SNP 1474	GG	13	20	
	GA	0	0	
	AA	0	0	
	Allelic Frequency A (%)			
SNP 1551	GG	2	7	
	GC	8	7	
	CC	3	6	
	Allelic Frequency C (%)	53	47	2.9 (0.5–17.3)
SNP 1922	TT	13	5	
	TG	2	1	
	GG	0	0	
	Allelic Frequency G (%)	13		0.8 (0.05–10.5)
SNP 2072	CC	4	10	
	CT	7	3	
	TT	4	4	
	Allelic Frequency T (%)	50	32	3.9 (0.9–17.5)
SNP 2168	GG	3	11	
	GA	6	1	
	AA	4	5	
	Allelic Frequency A (%)	54	32	6.1 (1.2–31.2)

AML: acute myeloblastic leukaemia; OR: odds ratio; CI: confidence interval.

**Table 4:** Prediction of UTR-SNPs affecting mRNA folding structures.

	number of sequences								
distance	dist>10	dist>20	dist>30	dist>40	dist>50	dist>60	dist>70	dist>80	average

Control (min-max)	155 (148–162)	126 (119–137)	104 (97–115)	81 (74–99)	60 (49–82)	38 (30–61)	23 (16–32)	8 (3–12)	20.30 (19.27–23.39)
cancer (n = 358)	179	136	115	94	77	52	20	4	22.85
cancer (permutation positive pool, n = 92)	49	39	32	27	22	17	7	2	25.24*

'Control': average of experiments with UTR-SNPs not associated with cancer phenotype (n = 358, 10 independent control trials). Results were statistically analyzed using the two sample t-test ('*', *p *< 0.05).

**Table 5:** Putative UTR regulatory elements affected by cancer-associated UTR-SNPs.

	UTR-SNPs Ref (n = 20,304)	UTR-SNPs Var (n = 20,304)	CANCER Ref (n = 358)	CANCER Var (n = 358)	CANCER Ref (permutation pool, n = 92)	CANCER Var (permutation pool, n = 92)
**Total IRES**	5766	5802	92	94	20	20
Gained		625		11		3
Lost		589		9		3
**Total 15-LOX-DICE**	1788	1744	37	36	8	8
Gained		90		2		
Lost		134		3		
**Total TOP**	491	490	11	10	5	5
Gained		1				
Lost		2		1		
**Total K-Box**	287	287	3	3	0	0
Gained		16				
Lost		16				
**Total GY-Box**	174	168	5	5	0	0
Gained		9				
Lost		15				
**Total Brd-Box**	111	116	2	1	1	0
Gained		13				
Lost		8		1		1
**Total ADH-DRE**	44	50	1	1	1	1
Gained		6				
Lost		0				
**Total CPE**	26	30	1	1	0	0
Gained		12				
Lost		1				
**Total SECIS-2**	12	10	1	0	1	0
Gained		1				
Lost		3		1		1

The results identify *cis*-regulatory elements located in the immediate vicinity of or at the UTR-SNP sites. UTR regulatory elements can be 'gained' or 'lost' when reference allele sequences are modified by cancer-associated SNPs.
