# Supplementary figures and images for: In silico whole-genome screening for cancer-related single-nucleotide polymorphisms located in human mRNA untranslated regions
Source: BMC Genomics. 2007 Jan 3;8:2. doi: 10.1186/1471-2164-8-2 (PMC1774567; doi:10.1186/1471-2164-8-2)

**A**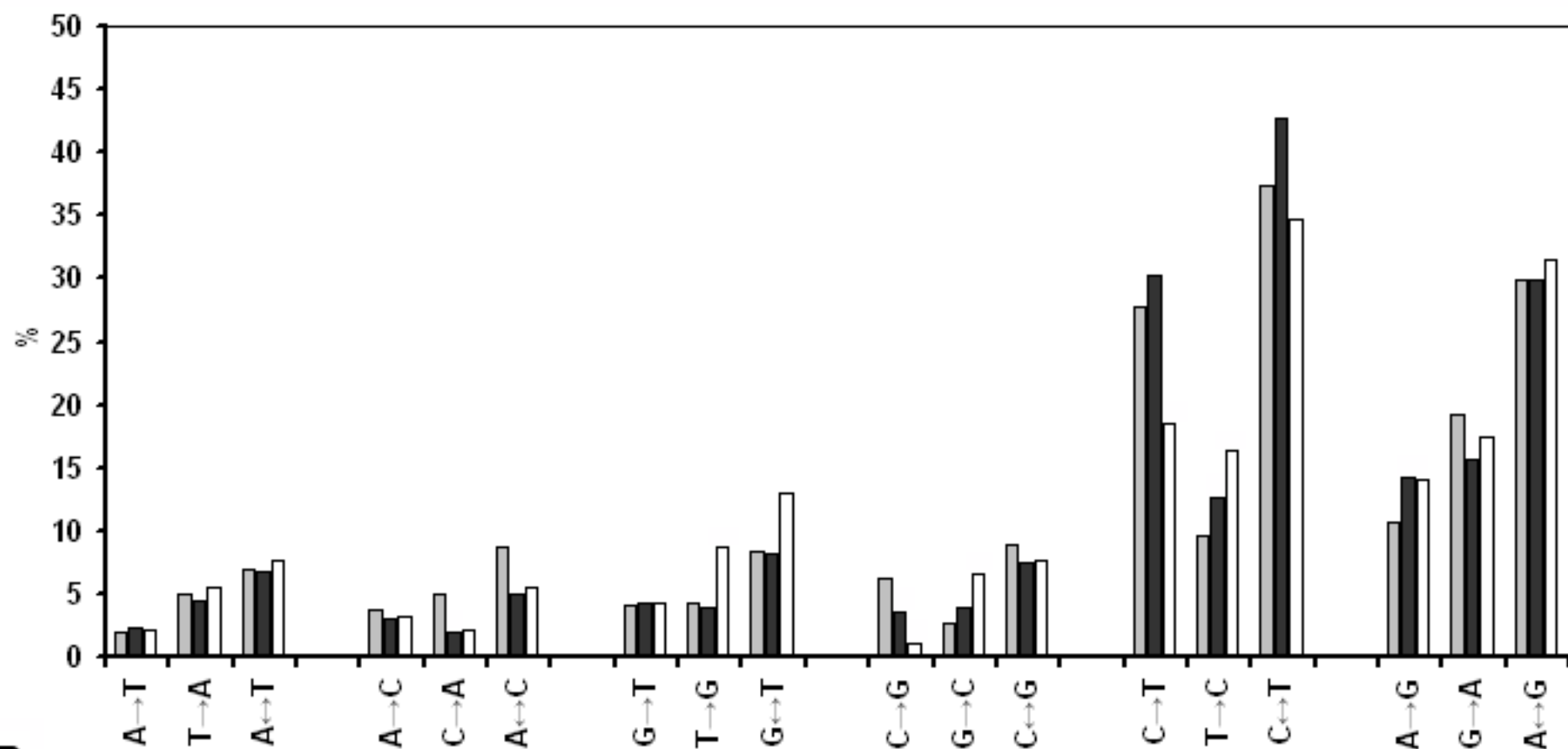**B**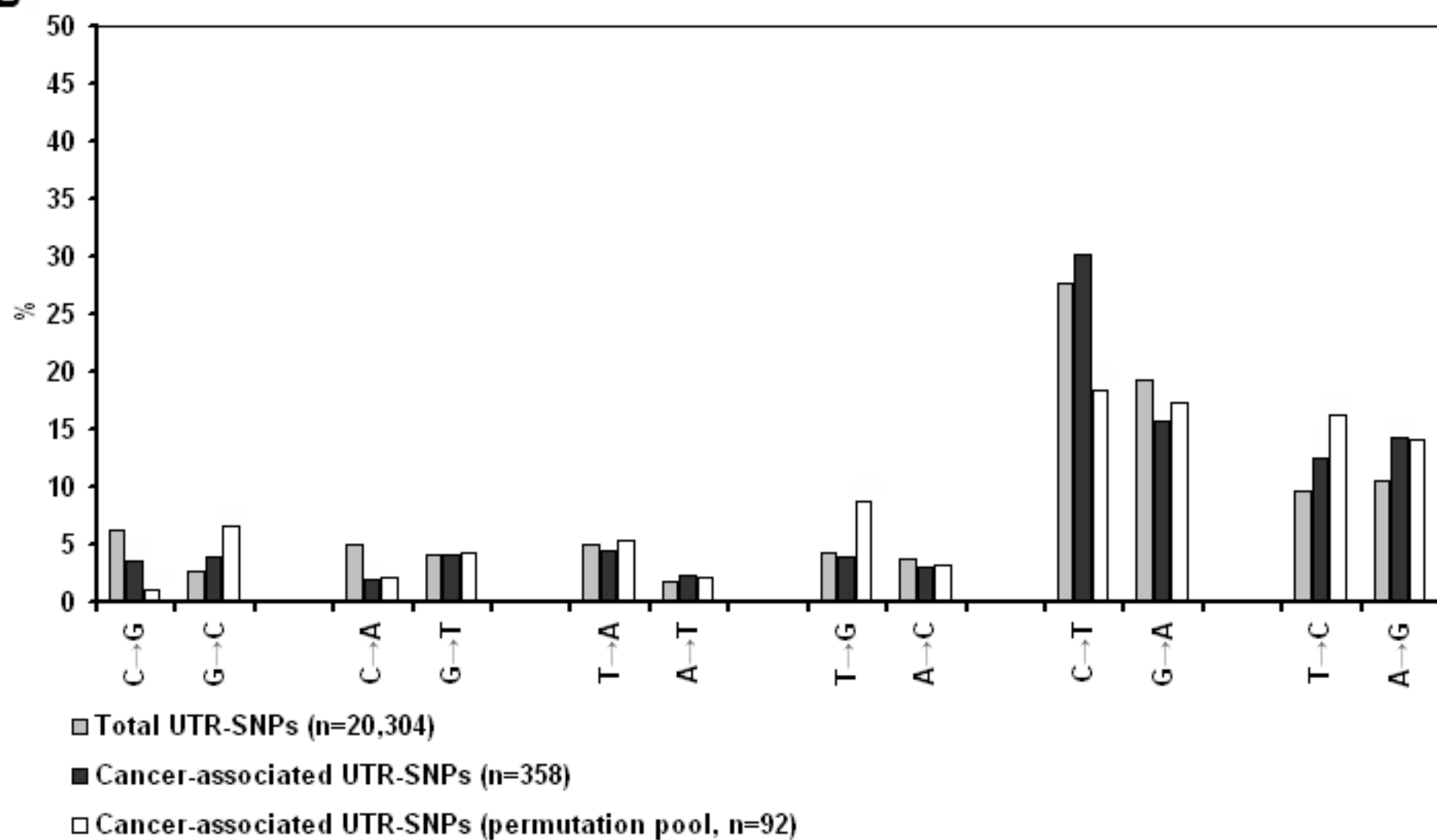

Supplement: Additional File 4 — Distribution of the different types of simple substitution SNPs (graphical representation). (A) Substitutional patterns observed among UTR-SNPs. Transition rates were 67.2 % in the complete dataset of UTR-SNPs, 72.6 % in the total pool of cancer-associated UTR-SNPs, and 66.3 % in the subset of UTR-SNPs which were positive after the resampling procedure. Of the 358 cancer-associated UTR-SNPs, 260 were transition events while 298 were transversion events. When considering the 92 UTR-SNPs positive after the resampling procedure, 61 were transition events and 31 were transversion events. (B) The proportions for each pair of complementary substitutions are graphed next to each other for ease of comparison. Student t-test not significant (p > 0.05). [file 1471-2164-8-2-S4.pdf]
